# A Digital Intervention for Capturing Real-Time Health Data for Epilepsy Seizure Forecasting: Protocol for the ATMOSPHERE Study

**DOI:** 10.2196/85993

**Published:** 2026-03-20

**Authors:** Lauren Thompson, Emily Nielsen, Emily E V Quilter, Amelia Slay, Marceli Wac, Matthew Wragg, Samuel Downes, Phil Tittensor, Leandro Junges, Liz Stuart, Rosie Charles, Peter Kissack, Yasser Qureshi, Amberly Brigden

**Affiliations:** 1 School of Engineering Mathematics and Technology University of Bristol Bristol United Kingdom; 2 Colinear Software Ltd Bristol United Kingdom; 3 The Royal Wolverhampton NHS Trust Wolverhampton United Kingdom; 4 Centre for Systems Modelling and Quantitative Biomedicine University of Birmingham Birmingham United Kingdom; 5 School of Computing Ulster University Belfast United Kingdom; 6 Neuronostics Bristol United Kingdom; 7 School of Mathematics University of Birmingham Birmingham United Kingdom; 8 School of Engineering University of Warwick Coventry United Kingdom

**Keywords:** epilepsy, seizure forecasting, data science, artificial intelligence, machine learning, data collection technology

## Abstract

**Background:**

Epilepsy is a chronic neurological disorder marked by recurrent and apparently unpredictable seizures and associated with premature death, injury, and diminished quality of life. The unpredictability of seizures is a major concern for people with epilepsy. Thus, developing tools for seizure prediction is a research priority. The Artificial Intelligence to Optimise Seizure Prediction to Empower People With Epilepsy (ATMOSPHERE) project focuses on the development and evaluation of seizure forecasting technology involving mobile technology and machine learning to provide personalized seizure forecasting (risk of seizure in the near future). The project is informed by complex intervention frameworks, which recommend phases of development, feasibility study, clinical evaluation, and implementation.

**Objective:**

Objective 1 aims to conduct a feasibility study to test and refine the trial methods for a future clinical trial. Objective 2 aims to test and refine the data collection technology, considering usability and technical performance. Objective 3 aims to collect longitudinal data on seizures and their precipitants to refine seizure forecasting.

**Methods:**

This study is a single-arm, mixed methods feasibility study, testing a prototype of the data collection technology, with phase 2 testing a minimum viable product. In total, 60 participants will be recruited via specialist National Health Service epilepsy clinics. Inclusion criteria are adults with epilepsy, experiencing seizures twice per month, able to consent, and engage with technology. Clinicians will screen and gain consent to contact, with researchers obtaining full consent. Participants will be invited to complete the following study procedures: (1) onboarding, (2) use the data collection technology (phase 1 or 2) in their lived context for up to 6 months, (3) complete patient-reported outcome measures and capture clinical-reported outcome measures at baseline and 3 months, and (4) complete a qualitative interview exploring their views of the data collection technology. A study flow diagram will report recruitment rates (outcome 1), diversity of the recruited sample (outcome 2), barriers and facilitators to recruitment (outcome 3), retention rates (outcome 4), and barriers and facilitators to retention (outcome 5). To assess the data collection technology, quantitative technology use data and qualitative interview data will be analyzed to assess usability (outcome 6) and technical performance (outcome 7) of the data collection technology. These outcomes will inform iterative minimum viable product development and testing cycles with stakeholders.

**Results:**

Recruitment is planned to begin in quarter 1 of 2026, with data collection expected to be completed by quarter 2 of 2027. Data analysis will take place during quarter 3, and the results will be published in quarter 4.

**Conclusions:**

This project aims to improve clinical outcomes for people with epilepsy through seizure forecasting technology. To evaluate clinical outcomes, robust trial methodology is critical. This feasibility study will optimize methods for a future full-scale clinical trial as well as refine the seizure forecasting intervention.

**International Registered Report Identifier (IRRID):**

PRR1-10.2196/85993

## Introduction

### Background

Epilepsy is a chronic neurological disorder, which is characterized by recurrent and unpredictable seizures [1]. Although there have been significant advancements in treatment, up to one-third of people with epilepsy still experience recurrent seizures [2]. Epilepsy has a significant impact on the well-being and quality of life of people with epilepsy, in which fear and the unpredictability of seizures are considered greatly debilitating characteristics of the condition [3,4], with the unpredictability of seizures being identified as having the greatest impact on the lives of people with epilepsy [5]. To address the problem of unpredictability, technology to enable the prediction of seizures has been identified as a research priority by the UK Epilepsy Priority Setting Partnership [6]. To tackle this issue, there is a growing body of research investigating the potential for technology to provide seizure forecasts [7]. This technology holds promise for helping people with epilepsy take proactive steps to reduce the risk of injury and lessen the psychological burden caused by the unpredictability of seizures [8].

Current technologies for epilepsy have primarily focused on detection (identifying a seizure that is already happening) rather than predicting or forecasting future seizures. Electroencephalography (EEG) remains the leading method for monitoring brain signals to detect seizure activity. However, it is not well-suited for seizure forecasting, as EEG is impractical for long-term use outside clinical settings [9]. Ultra–long-term EEG monitoring is an alternative, which allows continuous EEG data capture outside clinical settings and in individuals’ real-world context. However, ultra–long-term EEG monitoring requires surgical implantation, and the associated risks and costs may limit its accessibility for many users [10]. Less invasive alternatives such as scalp EEG are available; however, their adoption is also limited due to low acceptance among users [4].

With the increasing prevalence of wearable technologies such as smartwatches [11], there is significant potential for their application in epilepsy management and seizure forecasting. These devices, which are already embedded in many individuals’ daily routines, offer a convenient, noninvasive means of continuously capturing real-time physiological and behavioral data [12]. The Empatica E4 wristband has shown to be acceptable to users for epilepsy management and exhibits promise for seizure detection [9,13]. Advancements in machine learning and artificial intelligence have enabled the use of physiological data collected from wrist-worn devices to inform artificial intelligence algorithms for seizure forecasting, which have shown promising results [14,15]. However, despite the value provided by prior work, there remain limitations such as a narrow scope focusing only on circadian cycles and phases [16] or challenges with accuracy and generalizability [17].

The ATMOSPHERE (Artificial Intelligence to Optimise Seizure Prediction to Empower People With Epilepsy) project [18] aims to develop a seizure forecasting system using accessible data collection technology and machine learning. The data collection technology collects real-time data on evidence-based seizure precipitants (or triggers), for example, sleep quality, stress, and medication adherence [19,20]. These data are collected using sensors on a smartwatch and self-report surveys via a companion app using ecological momentary assessment (EMA) [21]. The system then uses machine learning for predictive analytics, creating an individualized risk score for the likelihood of seizures in the near future, which is presented to users through the companion app. The data collection technology was co-designed with clinicians and people with epilepsy (21 consultations). Their input informed the iterative design of the data collection technology used to collect the real-time data underpinning the seizure forecasting system. The resultant prototype was then usability tested and refined through a mixed methods, in-the-wild [15], longitudinal deployment study (n=14) [18]. In parallel, the seizure forecasting technology based on machine learning algorithms is in development. The development of the seizure forecasting system has been informed by state-of-the-art work in human-computer interaction and data science. The ultimate aim of the technology is to improve clinical outcomes for people with epilepsy. To determine this, the complex digital health intervention will need to be evaluated as part of a fully powered clinical trial investigating the clinical and cost-effectiveness of the technology.

A complex health intervention is defined by the UK Medical Research Council (MRC) as an intervention that has several interacting components or is dependent on the behaviors of those delivering and receiving the intervention, has a range of possible outcomes, or requires tailoring to different contexts and settings [22]. The MRC and the National Institute for Health and Care Research have developed a framework to provide guidance on complex health intervention development, recommending an approach of moving through 4 distinct phases: from development or identification of the intervention, to feasibility, then evaluation, and finally to the implementation phase [23]. The feasibility phase is a critical component, used to test trial methods on a small scale to assess whether a future, fully powered clinical trial can be undertaken [24]. Recruitment and retention of participants are typically the major challenges in the delivery of clinical trials [25,26], and mixed methods data during feasibility studies can be used to identify issues and optimize procedures to maximize these elements. In relation to recruitment, clinical trials need to recruit both a sufficient sample size and also sufficient diversity, for example, in relation to socioeconomic status, ethnicity, and age since a lack of diversity can exacerbate health inequalities [27]. Therefore, it is important to identify and reduce any modifiable barriers to participation, with particular attention to inclusive procedures for those groups typically underrepresented in health research [28]. With respect to retention and completion of outcome measures, it is crucial to avoid issues that lead to underpowered studies, as these can introduce bias in the interpretation of treatment effects. This depends on the extent of, and underlying reasons for, missing data, and how it is distributed across trial arms [29]. Therefore, it is essential to monitor attrition and completeness of the outcome measures (referring to the availability of data for specific measures or items) across arms of the trial. This allows for the assessment of retention and completion rates, as well as the identification of reasons for any missing data, which can inform potential strategies to address barriers.

In addition to optimizing trial procedures, the MRC-National Institute for Health and Care Research complex intervention framework recognizes that the feasibility phase can be used to optimize the intervention. Mixed methods data, such as quantitative use data and qualitative data on user experience, can inform intervention refinement to enhance usability and the clinical utility of the system. Further to usability refinement, the Clinical Trials Transformation Initiative recommends that feasibility studies should be used to assess, and if needed, correct technical performance, assessing elements such as issue-related data quality, unanticipated challenges, and weaknesses of the selected system [30].

### Aims and Objectives

Building on formative complex intervention development work, this study focuses on the second phase defined as feasibility testing. Initially, this single-arm, single-site feasibility study aims to test and refine the trial methods that will be used within a future fully powered clinical trial of clinical effectiveness. Second, it aims to test and refine the data collection technology. This is the component of the intervention designed to capture the real-time data underpinning the ATMOSPHERE seizure forecasting system.

The first objective is to assess and optimize trial processes. First, recruitment rates will be assessed (outcome 1) to understand the feasibility of recruiting people with epilepsy into the study. Specifically, we will record the number of people with epilepsy attending the clinic, screened by a clinician, eligible, providing consent to contact, and providing full informed consent. Second, we will assess the diversity of the recruited sample (outcome 2) by reporting the clinical and demographic characteristics of those consenting to participate. Third, the barriers and facilitators to recruitment will be explored with a focus on inclusive trial design for those underrepresented in health research (outcome 3). The number of participants leaving the study at each point of the recruitment phase as well as demographic characteristics and reason for withdrawal (where known) will be represented in the study flow diagram. If needed, Patient and Public Involvement (PPI) sessions with people with epilepsy and clinicians will be conducted to identify modifiable barriers and inform future recruitment strategies. Fourth, retention and outcome measure completion rates will be evaluated (outcome 4) by monitoring attrition and withdrawal rates throughout the study and reporting competition rates for each study procedure. Finally, barriers and facilitators to retention and outcome measure completion will be examined, with a focus on inclusive trial design (outcome 5). The number of participants who discontinue participation at each study procedure, together with their demographic characteristics and reasons for withdrawal (where known), will be summarized in the study flow diagram. Additional PPI sessions with people with epilepsy and clinicians will be undertaken to address modifiable barriers and inform future retention strategies.

The second objective is to assess and optimize the data collection technology of the seizure forecast system. Usability (outcome 6) will be assessed using both quantitative and qualitative methods. Quantitative technology use data, including onboarding rates and engagement metrics, will be analyzed to understand how participants interact with the system. In addition, qualitative interviews will be conducted to explore user experiences, perceptions of the technology, and suggestions for improvement. The technical performance (outcome 7) of the minimum viable product (MVP) and safety of the technology system (outcome 8) will be evaluated through a built-in function that allows users to report technical performance and safety issues within the app in real time. In addition, users’ perceptions of the app’s technical performance and safety issues encountered with the technology will be explored through qualitative interviews.

The third objective is to add the longitudinal data on seizures and seizure precipitants (data collected from the data collection technology) to the existing ATMOSPHERE dataset and share with the team developing the forecasting technology to refine and improve algorithmic performance.

## Methods

### Study Design

This study is a single-arm mixed methods feasibility study to assess and refine trial design (objective 1) and the data collection technology component (objective 2) to inform the future clinical evaluation of the seizure forecasting system. Recruitment is expected to begin in quarter 1 of 2026. The feasibility study will initially test a prototype of the data collection technology used to collect the data (phase 1), transitioning to testing an MVP of the data collection technology (phase 2). The data collected will be added to the existing dataset and used to refine the seizure forecasting algorithm (objective 3). This study protocol is reported in accordance with the CONSORT (Consolidated Standards of Reporting Trials) guidelines (see checklists in Multimedia Appendices 1 and 2).

### Participants and Recruitment

#### Overview

Potential participants will be identified during routine clinical appointments by clinicians from specialist National Health Service epilepsy clinics operated by the Royal Wolverhampton NHS Trust (RWT). RWT provides neurology services to almost 1 million people. Its core services are based in a large, multicultural inner city with significant levels of socioeconomic deprivation. However, RWT is also contracted to provide neurology services to people living in Shropshire and parts of Powys in Wales. These are largely rural localities. The diverse populations will enable assessment of the intervention across a range of geographical areas, as well as ethnic and socioeconomic groups. Clinicians will screen each patient at the clinic against the inclusion or exclusion criteria, maintaining a screening log. For those who are eligible, the clinician will provide a brief introduction about the study, including a participant information sheet, and obtain oral consent (1) to share their contact details with the research team and (2) for a member of the research team to contact them to discuss their participation in the study (consent to contact). Oral consent to contact will be documented in clinical records and screening log.

Eligible participants will be contacted by a member of the research team who will share further information about the study and invite participants to ask any questions they may have. For those choosing to take part, they will be asked to complete an online consent form via Microsoft Forms.

#### Inclusion or Exclusion Criteria

Adults (aged 18 years and older) will be eligible for inclusion if they have a diagnosis of epilepsy; are likely to have a seizure at least twice per month (seizure data required for modeling); are fluent in English (to be able to engage with the technology and qualitative interview); can provide informed consent; have access to an email address; can wear a wrist wearable device (smartwatch), supplied as part of the study; can input data into a smartphone app; own a smartphone that can download apps from either the Apple Store or the Google Play Store (for phase 1); and own an iPhone that can download apps from the Apple Store (for phase 2).

Adults will be excluded if they have a major cardiac condition, have a diagnosis of a major psychiatric condition (defined as needing secondary care mental health services support), and have a diagnosis of functional dissociative seizures or reflex epilepsy.

The aim is to screen all participants attending the clinics, with all participants who are eligible being given initial information and invited to consent to be contacted. All participants who consent to contact will be offered a meeting with a researcher and an opportunity to consent to the study. This is to reduce bias in the study. Where possible, reasons for nonscreening, nonconsent to contact, and nonconsent will be recorded, so that recruitment processes can be monitored.

### Sample Size

Several potential positive outcomes are anticipated from the use of the seizure forecasting system. It is hypothesized that these include improved clinical outcomes, better seizure control, reduced seizure-related injuries, enhanced locus of control, improved quality of life, and better mental health. Additionally, a reduction in health care resource use, such as fewer acute injury-related admissions, is also expected.

We will engage with stakeholders (people with epilepsy and clinicians) to identify which of these outcomes should be designated as the primary outcome for evaluation. This decision will be made once the complete seizure forecasting system has been finalized and is ready for clinical evaluation. Following this, the sample size required for the full-scale evaluation will be calculated based on the minimal clinically important difference for the selected primary outcome, ensuring that the trial is adequately powered to detect a clinically meaningful effect.

For this feasibility study, the sample size was determined by the 3 objectives:

- Objective 1 includes exploring the rate of recruitment and the diversity of the sample recruited. This will be used to inform a fully powered trial, for example, guiding the selection of additional sites and the length of the trial. This feasibility study will be used to determine the time frame needed to recruit 60 participants and the diversity of this sample. A sample size of 60 was selected to meet the requirements of objectives 2 and 3 (see below) and aligned with the typical sample size and recommendations for feasibility studies [31,32].
- Objective 2 focuses on refining the data collection technology of the seizure forecasting system. To do this, purposeful sampling will be used to identify 9-12 participants to invite them to take part in qualitative interviews to explore their experiences of using the MVP of the technology and identify any usability issues. Sampling for diversity will take place, with a focus on including individuals with lower digital literacy and from lower socioeconomic status groups. This is to ensure that the data collection technology is designed to include the needs of these groups to reduce the risks of digital exclusion and exacerbations of health inequalities. A sample size of 9-12 is considered sufficient to identify most usability issues in qualitative usability testing [33], ensuring a rich understanding of user experience and potential areas for improvement.
- Objective 3 seeks to add the data collected to the existing dataset to support the optimization of the seizure forecasting system. As the forecasting will be personalized and the system will be tailored to individual participants, the focus will be on obtaining accurate and continuous longitudinal data from each participant.

### The Data Collection Technology

#### Overview

The technology for collecting real-time data underpinning the seizure forecasting system consists of a smartwatch and a companion smartphone app referred to as the data collection technology. Previous iterations of the smartphone app prototype have undergone co-design and usability testing [18]. In this study, participants will be given either version 1 (the prototype) or version 2 (the MVP) of the technology, depending on their point of entry into the study. The primary difference between the prototype and MVP is that the prototype uses a third-party app (Labfront) integrated with the smartwatch, whereas the MVP will be a novel smartphone app developed by the research team. Both versions will collect the same data points, as outlined in Table 1.

**Table 1:** Data collection protocol.

Data point		Labfront data collection schedule	MVP^a^ data collection schedule
**Passive data collection via Garmin device**			
	Sleep score^b^	Daily summary	Daily summary
	Sleep stage^b^	Continuous	Continuous
	Sleep respiration	Continuous, 1-minute sampling rate	Continuous
	Stress^b^	Continuous, 10-second sampling rate	Continuous
	Intensity minutes	Continuous, 15-minute sampling	Continuous
	Motion intensity	Continuous, 15-minute sampling	Continuous
	Body battery^b^	Continuous, 3-minute sampling rate	Continuous
	Active calories burned	Continuous, 15-minute sampling	Continuous
	Step count	Continuous, 1-minute sampling rate	Continuous
	Actigraphy	Continuous, 1-minute sampling rate	Continuous
	Heart rate	Continuous, 10-second sampling rate	Continuous
	Heart rate interval	Continuous, each beat	Continuous
	Respiration rate	Continuous, 3-minute sampling rate	Continuous
	Pulse oximetry blood oxygen saturation	Continuous, 3-minute sampling rate	Continuous
	Accelerometer	Continuous, 15-minute sampling rate	Continuous
**User-initiated^c^ data via smartphone app (EMA^d^)**			
	Seizure event (type, timing, duration, severity)	Twice daily notification^e^	A twice daily notification and ad hoc user-initiated
	Prodromal symptoms	Twice daily (notification)	Twice daily notification and ad hoc user-initiated
	Postictal symptoms	Up to twice daily (notification; optional if seizure event reported)	As required (if seizure event reported)
	Epilepsy medication use: routine medication	Once daily (notification)	Customized notification and ad hoc user-initiated
	Epilepsy medication use: emergency medication	Once daily (notification)	As required, ad hoc user-initiated
	Emotional states and emotional intensity	Twice daily (notification)	Twice daily (notification) and ad hoc user-initiated
	Sleep duration and quality	Once daily (notification)	Once daily (notification)
	Acute illness or infection	Twice daily (notification)	Twice daily notification and ad hoc user-initiated
	Menstrual cycle	One off series of questions (optional)	One off series of questions (optional), which can be updated
	Alcohol	N/A^f^	Ad hoc user-initiated
	Personalized trigger	N/A	Ad hoc user-initiated

^a^MVP: minimum viable product.

^b^Compound measures through the Garmin application programming interface.

^c^User-initiated: users can complete the EMA item any time within the app.

^d^EMA: ecological momentary assessment.

^e^Notification: smartphone notification to prompt the user to complete the EMA item.

^f^N/A: not available.

#### Phase 1: Prototype

**Figure 1:**
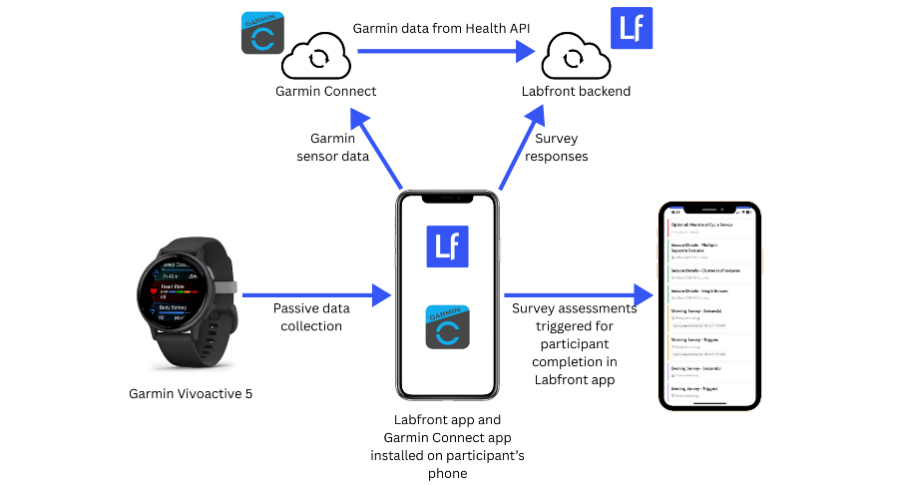
An overview of the phase 1 interactive prototype. API: application programming interface.

In phase 1, data collection will take place through a customized Labfront smartphone app installed on participants’ smartphones and connected via Bluetooth to a Garmin VivoActive 5 worn by participants (as outlined in Figure 1). Quantitative data captured passively through the Garmin wearable as well as compound measures, such as stress level and sleep score (Table 1), will be securely transmitted to the encrypted Labfront cloud by Garmin’s Health application programming interface. The use of Garmin’s application programming interface enables access to the raw data from the smartwatch. All participant data are anonymized before entering the Labfront system.

The EMA surveys that participants will receive in the Labfront app have been developed for participants to record data on seizures (timing, duration, type, and severity), seizure precipitants (triggers), prodromal symptoms, and postictal symptoms. EMA survey completion will be prompted twice daily by notification to the participant at a set morning and evening time. An optional menstrual cycle set-up survey may be completed at the start of participation. Completed surveys will be uploaded to the secure Labfront cloud, where they can be accessed by permissioned members of the core research team.

#### Phase 2: MVP

The design and development of the MVP were informed by earlier co-design and usability studies [18]. The anticipated development plan includes the creation of a custom-built smartphone app for phase 2, which will be developed using Swift, Apple’s native programming language for iOS. An agile software development methodology will be used, encompassing several iterative development and feedback cycles with stakeholders. Operating under a licensing agreement, the app will use the Garmin Health Standard Software Development Kit, which enables health and fitness data, such as heart rate and sleep, to be transferred from Garmin wearable devices to the app. Data being collected via the MVP are defined in Table 1. Quantitative data from the connected Garmin Vivoactive 5/6 device will be passively sent to the MVP app installed on an Apple iPhone. This will be relayed to an encrypted cloud database hosted in a secure digital environment in Amazon Web Services (AWS), independent of Garmin’s own services or databases. User self-reported data relating to EMA data defined in Table 1 will be similarly relayed to the same encrypted cloud database. Authentication to upload and download data from this database will be managed through AWS Cognito, which adopts the industry standard OAuth 2.0 protocol.

#### Algorithm Development

As part of this project, we are developing a seizure forecasting algorithm that uses physiological time series data collected from a smartwatch. The algorithm calculates time- and frequency-domain feature statistics, which are combined with seizure timings, obtained from the companion app to train and test a combination of machine learning models to extract seizure propensity scores in different time scales. The data collected via the data collection technology will be added to the existing ATMOSPHERE dataset and used to refine and advance the seizure forecasting algorithm.

### Study Procedures

**Figure 2:**
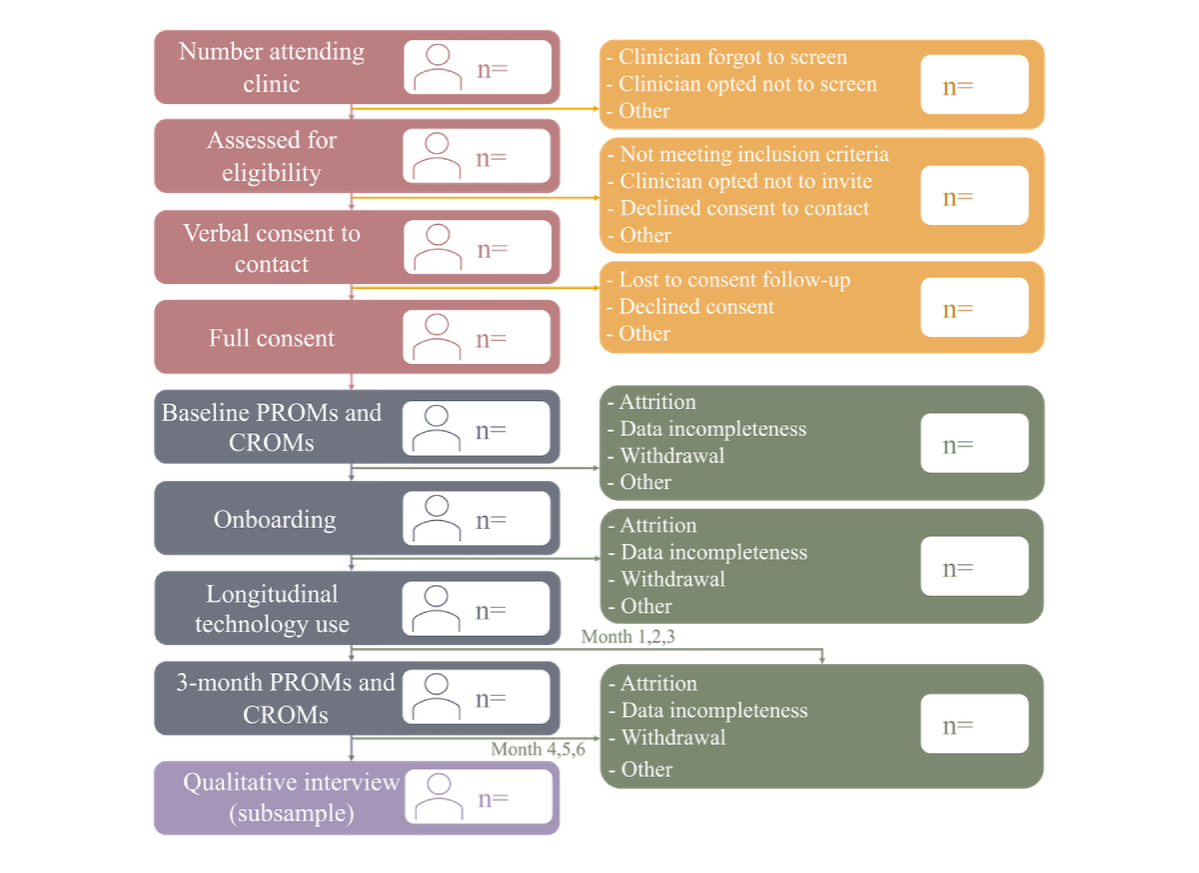
Study flow diagram showing participant flow through the study. Recruitment phases in pink and yellow, retention phases in blue and green, and a qualitative interview for a subgroup of participants in purple. CROM: clinician-reported outcome measure; PROM: patient-reported outcome measure.

Study procedures are illustrated in the template study flow diagram in Figure 2.

#### Procedure 1: Deployment of Prototypes

A pack containing a smartwatch and instructions for using the smartwatch and companion app will be sent to participants. An onboarding session will be held online with each participant to support the setup of the prototype. Participants will be invited to use the technology for up to 6 months. Each participant’s data will be reviewed monthly, and continuation of technology use for the following month will be contingent upon the participant wearing the smartwatch for some time and responding to the app questions on at least 21 days during the current month.

During the prototype deployment, incoming data will be monitored by the research team through dashboards generated within the Labfront software. Each week, participants will be sent an email summarizing their engagement with the prototype, including device wear time and survey completion. If missing data are noted, such as a survey completion rate falling below 80%, an in-app notification will be sent to participants reminding them to respond. If issues persist, follow-up via email will be conducted by the research team to offer troubleshooting support.

During the MVP deployment phase, incoming data will be monitored, and, if issues with use are identified, the MVP will be refined accordingly.

#### Procedure 2: Patient-Reported Outcome Measures and Clinician-Reported Outcome Measures

The following demographic and clinical information will be collected about participants at baseline by the clinical team based at the recruiting site. This will be taken from the clinical notes where possible, with additional data collected from the participant if it is not available in the notes: ethnicity, age, highest level of education completed, total annual household income, seizure type, and epilepsy medications and treatments.

Currently, there are no internationally accepted core outcomes for epilepsy, although one is under development [34]. The International Consortium for Health Outcomes Measurement (ICHOM) formed a global working group, including clinicians, people with epilepsy, and advocates, to define a standardized minimum set of outcomes for clinical practice [35]. The clinician-reported outcome measures (CROMs) and patient-reported outcome measures (PROMs) in this study capture these essential outcomes, including seizure- and non–seizure-related outcomes.

Participants will be asked to complete a series of PROMs at baseline and at 3 months. These include Patient-Reported Outcomes Measurement Information System (PROMIS) Cognitive Function Short Form [36], Patient Health Questionnaire (PHQ)-9 [37], Generalized Anxiety Disorder (GAD)-2 [38], PROMIS Sleep Disturbance—Short Form [39], and Quality Of Life in Epilepsy (QOLIE)-10 [40]. Participants will also be asked to complete a digital literacy scale, the Mobile Device Proficiency Questionnaire-16 [41], at baseline only.

A member of the clinical team will also complete a series of CROMs at baseline and at 3 months. These include: mortality; seizure frequency, seizure freedom, emergence of new seizure type, and seizure severity; emergency and unplanned health care services use; seizure control during pregnancy, pregnancy, and delivery complications; and memory.

Participants will have up to 1 month to complete the measures. These can be done either online or in paper format. An email reminder will be sent within the first 2 weeks after the expected completion date. If the measures remain incomplete after 2 weeks, a member of the research team will follow up by phone to assist with completion. Participants may be contacted by phone up to 3 times.

#### Procedure 3: Qualitative Interview and Usability Questionnaire (Phase 2 Only)

At the end of the technology deployment, participants who used version 2 of the prototype (MVP) will be invited to participate in a qualitative interview with a member of the research team. These interviews will be scheduled for up to 60 minutes and use a semistructured topic guide (Multimedia Appendix 3) designed to explore participants’ views and experiences of using the technology.

Participants will also be asked to complete the mHealth App Usability Questionnaire (MAUQ), an 18-item questionnaire, which consists of 3 subscales that focus on ease of use (5 items), interface and satisfaction (7 items), and usefulness (6 items), rated on a 7-point Likert scale (1=strongly disagree to 7=strongly agree) [42].

### Outcome Measures and Planned Analyses

#### Objective 1: Feasibility Outcomes Measures and Analysis: Trial Procedures

##### Overview

Quantitative and qualitative data will be used to assess and optimize the trial procedures and intervention component. Using the screening logs, consent outcomes, and the number of participants completing study procedures (onboarding, use of the technology longitudinally, PROMs at baseline and 3 months, and qualitative interview), we will create a study flow diagram to present descriptive statistics on the participant flow through the study, as per Figure 2.

##### Outcome 1: Recruitment Rates

The number of participants at each recruitment phase will be represented in the study flow diagram, as illustrated in Figure 2. The feasibility of recruitment will be assessed through the following indicators: the number of patients (1) attending the clinic, (2) screened by a clinician, (3) eligible to participate, (4) providing consent to contact, and (5) providing full consent. To determine the feasibility to progress to a larger study and full trial, we expect to reach a minimum of 70% of the recruitment target.

##### Outcome 2: Diversity of Recruited Sample

The clinical and demographic characteristics of those consenting to the study will be reported to evaluate the diversity of the recruited sample. Characteristics will include seizure type, age, gender, ethnicity, socioeconomic status, and digital literacy. This will help assess whether the recruitment approach was effective in enrolling a diverse sample and be used to inform a purposive recruitment strategy for a full trial if necessary.

##### Outcome 3: Barriers and Facilitators to Recruitment, With a Focus on Inclusive Trial Design for Those Underrepresented in Health Research

###### Quantitative Descriptive Statistics

The number of participants leaving the study at each point of the recruitment phase, along with their demographic characteristics and reason for withdrawal (were known), will be represented in the study flow diagram (Figure 2).

###### Stakeholder Consultations

If issues are identified during the recruitment phase, we will engage with people with epilepsy and the key members of the clinical and research teams to identify and document any barriers or problems related to recruitment. In response, practical, context-specific solutions aimed at improving recruitment processes will be developed and implemented collaboratively with the clinical team. This iterative approach will ensure that emerging challenges are addressed promptly and that recruitment strategies remain acceptable and feasible within the clinical setting. A full trial is unlikely to be feasible if we do not reach 70% of our recruitment target, and the qualitative data suggest that recruitment cannot be improved any further.

##### Outcome 4: Retention

To assess participant retention, the number of participants remaining in the study at each study procedure will be reported in the study flow diagram (Figure 2). Specifically, the following will be reported: the number of participants who completed (1) onboarding, (2) the technology deployment phase (either phase 1 or 2 version) in their lived context for up to 6 months, (3) PROMs at baseline and 3 months, and (4) a qualitative interview exploring their views of the technology (conducted with a subsample of 9-12 participants). To determine the feasibility to progress to a larger study and full trial, we expect to reach 80% retention at follow-up.

##### Outcome 5: Barriers and Facilitators to Retention, With a Focus on Inclusive Trial Design for Those Underrepresented in Health Research

The number and demographics of participants who leave the study, along with reasons for leaving (where known), will also be reported. If issues are identified during the retention phase, we will conduct PPI sessions with people with epilepsy to identify possible solutions to any barriers. A full trial is unlikely to be feasible if retention is <80%, and the qualitative data suggest that retention cannot be improved any further.

#### Objective 2: Feasibility Outcomes Measures and Analysis: Refining the Intervention

##### Outcome 6: Usability

###### Quantitative Use Metrics

Usability will be assessed through descriptive statistics of wear time (assessed using the presence of heart rate data), proportion of participants who complete the scheduled questionnaires at each time point. These quantitative indicators will offer insight into the feasibility and acceptability of both the data collection technology and associated data collection procedures.

To determine feasibility to progress to a larger study and full trial, we will consider the study feasible if at least 80% (≥48/60) of participants achieve an average wear time of ≥19 hours per day across the study period and a total average score of 5 on the MAUQ. This threshold was informed by established usability benchmarks for the System Usability Scale, which has been shown to be strongly correlated with the MAUQ [42].

###### Quantitative User Experience (Phase 2 Only)

Usability will be assessed quantitatively by calculating the total and average scores of the MAUQ items.

###### Qualitative User Experience (Phase 2 Only)

Usability will also be assessed through qualitative interviews with participants who use the MVP version of the app. Interview data will be analyzed using thematic analysis to identify key themes related to the acceptability and feasibility of the app in its current form. In addition, tables of change will be used to systematically track suggested improvements and user feedback, highlighting specific areas where the app may require refinement. It is unlikely that a full trial will be feasible if the data suggest that the technology is not acceptable to users.

##### Outcomes 7 and 8: Technical Assessment and Safety Reporting (Phase 2 Only)

The number of issues reported by participants will be presented. The types of issues reported will be described following a content analysis of the issue reports. The study’s safety officer (PT; clinical lead) will review the issues reported and will determine whether the study should be stopped.

#### Objective 3: Refining the Algorithm Development

The longitudinal data on seizures and seizure precipitants (data collected from the data collection technology) will be added to the existing ATMOSPHERE dataset, shared with the modeling team to support the continued development of the seizure forecasting algorithm.

The modeling team will evaluate a combination of machine learning algorithms for seizure forecasting. Continuous physiological time series will be windowed (with segments ranging between 1 and 36 hours), and features will be calculated for each segment. Features will include statistical measures in the time domain (eg, mean, median, variance, skewness, and kurtosis) and the frequency domain (eg, mean frequency and peak frequency).

Features will be used to inform machine learning models to predict seizures in a period of time after each segment (forecasting window—ranging from 1 to 24 hours). Models will include 1-class support vector machine, isolation forest, and extreme gradient boosting. Model performance will be evaluated using well-established metrics such as accuracy, balanced accuracy, sensitivity, and specificity. We will also use a user-centered approach to evaluate performance, training the algorithm to minimize false positive or negative rates according to user preference. Model accuracy will be validated using a 5-fold cross-validation approach.

### Ethical Considerations

This study was reviewed and granted ethics approval by the South East Scotland NHS committee (REC reference: 25/SS/0116). Participants will be asked to provide informed consent by completing an online consent form administered through Microsoft Forms. There is a minor risk that participants will find the smartwatch device uncomfortable to wear. Participants will be made aware of this risk and that they can stop wearing the device at any time via the participant information sheet. If participants report finding the watch uncomfortable, an alternative soft watch strap will be offered as a replacement. There is a risk that participants will experience a seizure when in the presence of the research team, for example, during a qualitative interview. The research team has read an Epilepsy First Aid document written by the charity Epilepsy Action and provided by our Consultant Nurse for the Epilepsies. The research team will also have the contact details of a health care professional with sufficient expertise (such as an epilepsy nurse) with epilepsy in the event that additional support is needed. At the start of any meeting with participants, researchers will make a joint plan at the beginning to identify what the participant would like the researcher to do in the event of a seizure. Participants will be asked to share information about emotional states so there is a possibility that this could cause participants to reflect on negative states. Participants will be made fully aware of this and provided information about this procedure, including that they will be able to stop engaging in this procedure at any point. Participants are being asked to contribute time and effort to the study—the time and effort of wearing the smartwatch, using the smartphone app, engaging in qualitative interviews, and completing outcome measures. Participants will be made aware of this burden in the information sheet, and they will be free to withdraw from study procedures at any time. These will be stored on a separate server to research data so that they cannot be linked. Participants will be allocated a unique research ID, and all research data will be labeled with the research ID and no identifiable data. A Microsoft Excel file linking identifiable information (participants’ names) with their research ID will be maintained to enable pseudonymization. The Excel file will be stored securely within the University of Bristol environment, with restricted access to the research team and stored separately from research data. For qualitative research data, audio recordings of qualitative interviews will be deleted once transcription is complete. Transcripts will be pseudonymized with all personal or identifiable information redacted. Baseline characteristics, outcome measure forms (PROMs and CROMs), and MAUQ will be captured via Microsoft Forms. PROMs and CROMs will be entered into Microsoft Forms directly by participants and by the clinical team and will be accessed by members of the research team. The data collection system has been designed to be secure. Labfront is a data collection tool designed for academic researchers. It is a secure solution for collecting high-resolution physiological data from wearables and survey responses. Storage of data will be in the United States, meeting the appropriate security and privacy regulations and certifications. To ensure data privacy and protection, a Data Protection Impact Assessment was conducted, which aims to evaluate and minimize risks to the rights and freedoms of individuals’ personal data (aligned with General Data Protection Regulation) [43], which was approved by the information compliance manager and data protection officer at the university. The pseudonymized data will be shared with the modeling collaborators via the secure Research Data Storage Facility to ensure controlled and protected access. The information from the app will be hosted in the cloud using AWS. The development of this will follow industry standard methods of data handling, storage, and transfer, including data encryption on device and in transit using HTTPS, in accordance with the UK General Data Protection Regulation. At this stage, the Data Protection Impact Assessment will be renewed to reflect the respective changes to data flows and storage. Participants will be given approximately £50 (approximately US $68) in shopping vouchers per month that they are involved in the study, including a further £25 (approximately US $34) voucher for participating in a qualitative interview.

## Results

### Overview

Recruitment is planned to begin in quarter 1 of 2026, with data collection expected to be completed by quarter 2 of 2027. Data analysis will take place during quarter 3, and the results will be published in quarter 4.

### Dissemination of Findings

Feasibility outcomes (objectives 1 and 2) will be analyzed and written up for publication in quarter 3 of 2027. Objective 3 is to add the longitudinal data on seizures and seizure precipitants (data collected from the data collection technology, the smartwatch, and smartphone app) to the existing ATMOSPHERE dataset to optimize the machine learning seizure forecasting algorithm. The machine learning analysis will be reported separately. The outcomes of the feasibility study will be reported according to the CONSORT extension to pilot and feasibility trials [44,45].

The outcomes of this study will be disseminated through open-access peer-reviewed publications via research journals and conferences. Research findings will also be disseminated to people with epilepsy and public networks via mechanisms such as workshops, plain English summaries, and blog posts.

## Discussion

### Principal Findings

This feasibility study aims to lead to optimized methods for a full-scale clinical evaluation (objective 1) and a refined version of the mobile data collection component MVP (objective 2). In parallel to this study, the data science research and development will refine the seizure forecasting algorithm, which will be optimized through data from this feasibility study (objective 3). As such, this feasibility study is a critical step in finalizing the seizure forecasting technology and delivering a fully powered trial to determine clinical effectiveness.

### Strengths and Limitations

The study was designed to test trial procedures, including recruitment, retention, and outcome measures (objective 1). A key strength is the focus on optimizing the trial process to include individuals who are typically underrepresented in health research, such as those from socioeconomically disadvantaged backgrounds and ethnic minority groups [46]. At this initial single site, the representation of these groups will be monitored, and findings will directly inform the selection of additional sites for the full-scale clinical trial in order to improve diversity and reach. A limitation associated with objective 1 is that the methodology used in this smaller-scale study is likely to differ in several respects from the anticipated design of the full-scale trial, meaning that certain elements of the full trial will not have been feasibility-tested. Specifically, this study is being conducted at a single site using a single-arm design, whereas the full trial is expected to adopt a randomized controlled trial design. Additionally, outcome measures are being collected at baseline and at 3 months, while the full-scale trial is anticipated to involve a longer follow-up period (eg, up to 12 months). As a result, multisite recruitment, randomization procedures, and long-term outcome measurement will remain untested; however, it is typical for feasibility studies to use a simplified design. Furthermore, outcome measures in this study have been derived from the ICHOM clinical outcomes framework, as no research core outcome sets for epilepsy currently exist [35]. The Epilepsy Outcome Set for Effectiveness Trials (EPSET) project [34] is developing an epilepsy outcome set for effectiveness trials, which may become available in time for the fully powered trial. If so, the outcome measures will be amended to align with the EPSET Core Outcome Sets. This would result in a discrepancy between the outcome measures assessed during the feasibility study and those used in the larger trial. However, it is likely that ICHOM clinical outcomes and EPSET will be largely aligned; therefore, it is expected that the measures used in this study will likely still produce relevant feasibility insights. Finally, as the primary outcome will be determined upon finalization of the intervention, a power calculation for the full trial has not yet been conducted. However, this feasibility study will provide estimates of recruitment rates, which will inform realistic projections of timelines and the number of sites required to achieve the desired sample size once it is defined.

The study was also designed to refine the intervention (objective 2). A strength of this phase is that sampling for the qualitative usability testing has been structured to prioritize inclusivity. This is particularly important, given that underrepresented groups are at increased risk of digital exclusion and health inequalities [47], as well as experiencing a much higher burden of epilepsy [48]. Usability and technical performance are being assessed through both qualitative and quantitative data, providing insights into objective use across the full sample, alongside in-depth data on user perspectives and experiences.

Finally, this study is evaluating 1 component of the seizure forecasting technology, the data collection component. Valuable insights will be generated regarding various aspects of the technology, including the onboarding process, the design and functionality of the frontend interface, and the technical performance of the system. However, the final version of the technology will incorporate algorithm integration and model outputs within the backend. As such, further rounds of user testing, technical performance evaluation, and safety assessment will be required prior to the full-scale trial. Nonetheless, testing at this early stage is considered a strength, as it enables a more agile development process. Early learning about data collection challenges can occur in parallel with the ongoing development of the data science components and human-computer interaction elements.

### Conclusions

Seizure forecasting technology holds significant promise for improving clinical and cost-effectiveness outcomes for individuals living with epilepsy. This mixed methods feasibility study is designed to assess the feasibility of the proposed trial procedures and to refine the forecasting technology in a real-world setting. Insights gained from this study will be critical for optimizing the design and implementation of a future full-scale clinical trial, ensuring that it is both methodologically robust and patient-centered.

## Appendix

Multimedia Appendix 1 CONSORT checklist.

Multimedia Appendix 2 CONSORT abstract checklist.

Multimedia Appendix 3 Interview topic guide.

## Abbreviations

ATMOSPHERE: Artificial Intelligence to Optimise Seizure Prediction to Empower People With Epilepsy
AWS: Amazon Web Services
CONSORT: Consolidated Standards of Reporting Trials
CROM: clinician-reported outcome measure
EEG: electroencephalography
EMA: ecological momentary assessment
EPSET: Epilepsy Outcome Set for Effectiveness Trials
ICHOM: International Consortium for Health Outcomes Measurement
MAUQ: mHealth App Usability Questionnaire
MRC: Medical Research Council
MVP: minimum viable product
PPI: Patient and Public Involvement
PROM: patient-reported outcome measure
RWT: Royal Wolverhampton NHS Trust

## Data Availability

The datasets generated or analyzed during this study are available from the corresponding author on reasonable request..
