# Supplementary material for: A Digital Intervention for Capturing Real-Time Health Data for Epilepsy Seizure Forecasting: Protocol for the ATMOSPHERE Study
Source: JMIR Res Protoc. 2026 Mar 20;15:e85993. doi: 10.2196/85993 (PMC13049396; doi:10.2196/85993)
Supplement: Multimedia Appendix 2 [file resprot_v15i1e85993_app2.pdf]

CONSORT 2010 checklist of information to include when reporting a pilot or feasibility randomized trial in a journal or conference abstract

Item	Description	Reported on line number
Title	Identification of study as randomised pilot or feasibility trial	10
Authors *	Contact details for the corresponding author	N/A
Trial design	Description of pilot trial design (eg, parallel, cluster)	14
Methods		
Participants	Eligibility criteria for participants and the settings where the pilot trial was conducted	17
Interventions	Interventions intended for each group	19-23
Objective	Specific objectives of the pilot trial	10-14
Outcome	Prespecified assessment or measurement to address the pilot trial objectives**	24-29
Randomization	How participants were allocated to interventions	N/A
Blinding (masking)	Whether or not participants, care givers, and those assessing the outcomes were blinded to group assignment	N/A
Results		
Numbers randomized	Number of participants screened and randomised to each group for the pilot trial objectives**	N/A
Recruitment	Trial status†	17
Numbers analysed	Number of participants analysed in each group for the pilot objectives**	N/A
Outcome	Results for the pilot objectives, including any expressions of uncertainty**	N/A
Harms	Important adverse events or side effects	N/A
Conclusions	General interpretation of the results of pilot trial and their implications for the future definitive trial	N/A
Trial registration	Registration number for pilot trial and name of trial register	N/A
Funding	Source of funding for pilot trial	N

Citation: Eldridge SM, Chan CL, Campbell MJ, Bond CM, Hopewell S, Thabane L, et al. CONSORT 2010 statement: extension to randomised pilot and feasibility trials. BMJ. 2016;355.

**this item is specific to conference abstracts*

***Space permitting, list all pilot trial objectives and give the results for each. Otherwise, report those that are a priori agreed as the most important to the decision to proceed with the future definitive RCT.*

†For conference abstracts.
