# Supplementary material for: A Digital Intervention for Capturing Real-Time Health Data for Epilepsy Seizure Forecasting: Protocol for the ATMOSPHERE Study
Source: JMIR Res Protoc. 2026 Mar 20;15:e85993. doi: 10.2196/85993 (PMC13049396; doi:10.2196/85993)
Supplement: Multimedia Appendix 3 [file resprot_v15i1e85993_app3.docx]

Usability Interview Topic Guide

**Section 1: Exploring Participants' Experiences of Using the Prototype**

*General Experience*

- Can you describe your overall experience using the Garmin watch and app over the past four weeks?
- How did you integrate the wearable device and answering the surveys into your daily routine?
  - Were there any challenges?

*Wearable Device (Garmin)*

- How comfortable was the Garmin watch to wear throughout the day and night?
- Did you encounter any issues with wearing the device continuously?
  - If so, please describe.

*Smartphone Application*

- How easy or difficult was it to navigate the app and input the required data?
- Were there any specific items or questions in the app that you found burdensome or particularly helpful?
- How did the notifications and prompts from the app feel in terms of frequency and timing?
  - Did you find them helpful/ intrusive?

*Specific Items*

- Are there any items or questions in the app that you would suggest adding or removing?
  - Why?
- How did you feel about the wording of the questions and prompts in the app?
  - Was anything unclear or confusing?

*Socio-technical Space*

- How did using the wearable device and app fit into your overall daily life and social context?
- Did you experience any social challenges or benefits from using the technology in public or around others?

**Section 2: Exploring Participants’ Views on Improvement**

*Smartphone Application*

- How could the app be improved in terms of user interface and functionality?
- Are there any additional features, capabilities or questions you think the app should have?
- How can the app’s notifications and prompts be adjusted to be more effective?
- Would you like to track any other potential seizure triggers that aren’t currently being tracked/recorded by the app?

*Wearable Device (Garmin)*

- What improvements or changes would you suggest for the Garmin watch to make it more user-friendly?
- Different wearable?

Are there any additional features you think would be useful to include in the device?

*Data visualisation*

- Would you find visual representations of the data collected by the app and Garmin watch helpful?
  - If so, what type of information would you like to see visualised? (e.g., seizure frequency, sleep patterns, stress levels)
- How would you like this information to be presented? (e.g., graphs, charts, timelines)
- Do you have any suggestions for making these visualisations more accessible and easier to understand?

**Section 3:**

- Did you experience any technical difficulties (e.g. crashes) when using the wearable device or apps?
- Did you experience any other issues (e.g. safety related) associated with using the wearable device or app?

**Section 4: Additional Comments and Suggestions**

- Is there anything else you would like to add about your experience using the wearable device and the app?
- Do you have any additional suggestions or feedback that we haven’t covered?
